# The calcium stored in the sarcoplasmic reticulum acts as a safety mechanism in rainbow trout heart

**DOI:** 10.1152/ajpregu.00127.2014

**Published:** 2014-11-05

**Authors:** Caroline Cros, Laurent Sallé, Daniel E. Warren, Holly A. Shiels, Fabien Brette

**Affiliations:** ^1^Faculty of Life Sciences, The University of Manchester, Core Technology Facility, Manchester, United Kingdom; and; ^2^EA 4650, Université de Caen, Caen Cedex, France

**Keywords:** calcium current, calcium-induced calcium release, excitation-contraction coupling, cardiomyocyte, fish

## Abstract

Cardiomyocyte contraction depends on rapid changes in intracellular Ca^2+^. In mammals, Ca^2+^ influx as L-type Ca^2+^ current (*I*_Ca_) triggers the release of Ca^2+^ from sarcoplasmic reticulum (SR) and Ca^2+^-induced Ca^2+^ release (CICR) is critical for excitation-contraction coupling. In fish, the relative contribution of external and internal Ca^2+^ is unclear. Here, we characterized the role of *I*_Ca_ to trigger SR Ca^2+^ release in rainbow trout ventricular myocytes using *I*_Ca_ regulation by Ca^2+^ as an index of CICR. *I*_Ca_ was recorded with a slow (EGTA) or fast (BAPTA) Ca^2+^ chelator in control and isoproterenol conditions. In the absence of β-adrenergic stimulation, the rate of *I*_Ca_ inactivation was not significantly different in EGTA and BAPTA (27.1 ± 1.8 vs. 30.3 ± 2.4 ms), whereas with isoproterenol (1 μM), inactivation was significantly faster with EGTA (11.6 ± 1.7 vs. 27.3 ± 1.6 ms). When barium was the charge carrier, inactivation was significantly slower in both conditions (61.9 ± 6.1 vs. 68.0 ± 8.7 ms, control, isoproterenol). Quantification revealed that without isoproterenol, only 39% of *I*_Ca_ inactivation was due to Ca^2+^, while with isoproterenol, inactivation was Ca^2+^-dependent (∼65%) and highly reliant on SR Ca^2+^ (∼46%). Thus, SR Ca^2+^ is not released in basal conditions, and *I*_Ca_ is the main trigger of contraction, whereas during a stress response, SR Ca^2+^ is an important source of cytosolic Ca^2+^. This was not attributed to differences in SR Ca^2+^ load because caffeine-induced transients were not different in both conditions. Therefore, Ca^2+^ stored in SR of trout cardiomyocytes may act as a safety mechanism, allowing greater contraction when higher contractility is required, such as stress or exercise.

the contractile activity of vertebrate cardiomyocytes depends on rapid changes in free intracellular Ca^2+^ concentration [Ca^2+^]_i_, which is controlled by a fine balance of ion channels and exchangers during the excitation-contraction (EC) coupling process (8). Upon the cardiac action potential (AP), Ca^2+^ influx through the L-type Ca^2+^ channels (LTCCs) triggers the release of additional Ca^2+^ (Ca^2+^-induced Ca^2+^ release, CICR) (24) from closely apposed clusters of sarcoplasmic reticulum (SR) Ca^2+^ release channels or ryanodine receptors (RyRs) that amplifies the Ca^2+^ current (*I*_Ca_). The resulting net increase in global systolic Ca^2+^ transient, formed by the spatial and temporal sum of those local Ca^2+^ events, subsequently activates the contractile machinery within the cardiomyocyte (19). An assortment of pumps and exchangers then move Ca^2+^ back to internal SR stores and out of the cytosol, after which the EC coupling process starts anew.

To limit Ca^2+^ entry during each heartbeat and prevent cell Ca^2+^ overload, LTCCs undergo inactivation, induced by both Ca^2+^ and voltage [Ca^2+^-dependent inactivation, CDI and voltage-dependent inactivation, VDI] (31). In mammals, CDI is the predominant mechanism of LTCC inactivation [e.g., in rat (1); in guinea pig (33) or in rabbit (40)], is induced by both sarcolemmal and SR Ca^2+^ (35, 42, 49) (together forming total CDI), and is an important negative feedback regulator that also regulates SR Ca^2+^ load and modulates the AP duration (2). Another modulation of the Ca^2+^ channels includes Ca^2+^-dependent facilitation (CDF) that potentiates LTCC Ca^2+^ influx during repeated activity and contributes to increasing the force-frequency relationship of some mammalian hearts during exercise (3).

In contrast to mammals, our understanding of cardiac Ca^2+^ cycling and Ca^2+^ channel regulation in fish cardiomyocytes is incomplete, and quantitative data are lacking (48). Despite numerous studies showing that the fish myocardium contains all the cellular components necessary for the EC coupling found in mammalian heart, it is still unclear whether sarcolemmal Ca^2+^ influx alone is sufficient to evoke contraction (e.g., 54, 55) or whether internal Ca^2+^, stored in the SR, plays an active role (58). This study addresses this point.

One approach to characterize the role of SR Ca^2+^ release in cardiomyocytes is to investigate the inactivation phase of LTCCs. Using two Ca^2+^ buffers with different kinetics of chelation (EGTA, slow and BAPTA, fast), it is possible to discriminate between action of CICR from the SR upon *I*_Ca_ or not. This approach was first developed in neurons to characterize neurotransmitter secretion (36). In cardiomyocytes, it has been shown by numerous laboratories that EGTA allows Ca^2+^ to be released from the SR at the local level, whereas BAPTA prevents it (16). To further differentiate the portion of LTCC inactivation due to VDI from CDI, Ca^2+^ can be substituted to Ba^2+^ as the charge carrier (in absence of Ca^2+^, CDI is abolished), and the Ba^2+^ current via LTCC (*I*_Ba_) is used as a measure of VDI (29). Thus, we have applied this well-accepted method for the first time in fish ventricular cardiomyocytes using rainbow trout (*Oncorhyncus mykiss*) as an experimental model to *1*) quantify *I*_Ca_ inactivation and *2*) investigate whether *I*_Ca_ can trigger SR Ca^2+^ release. In addition, we have recorded caffeine-induced systolic Ca^2+^ transients to ascertain the presence of Ca^2+^ in the SR of fish cardiomyocytes.

We show that in control conditions *I*_Ca_ does not induce SR Ca^2+^ release and that external Ca^2+^ is the main trigger of cardiac contraction. In contrast, during β-adrenergic stimulation, CICR occurs and Ca^2+^ released from the SR is a major source of Ca^2+^ flux into the cytosol and of the intracellular Ca^2+^ transient that activates rainbow trout cardiomyocytes contraction. We propose that the Ca^2+^ stored in the SR of rainbow trout myocytes may act as a reserve and is released only when extra Ca^2+^ is required for greater cell contraction to maintain or enhance cardiac function, such as during stress.

## MATERIALS AND METHODS

### 

#### Fish origin and care.

Rainbow trout (*Oncorhyncus mykiss*) were obtained from Chirk Fish Farm (Wrexham, UK). Fish were held in freshwater tanks at 12 ± 1°C with a 12:12-h light-dark cycle and fed with commercial fish pellets. All procedures were in accordance with local animal handling protocols and adhere to UK Home Office legislation.

#### Cardiomyocytes isolation.

Cardiomyocytes were isolated by enzymatic dissociation, as previously described (44, 55). Rainbow trout were humanely killed, after which the heart was carefully excised. The heart was cannulated through the bulbus arteriosus into the ventricle and perfused with a Ca^2+^-free isolation solution (see *Solutions*). After 10 min, the isolation solution was supplemented with collagenase (type IA, 0.4 mg/ml), trypsin (type IX-S, 0.2 mg/ml), and BSA (0.7 mg/ml), and the perfusion was continued for another 15 min. Next, the heart was cut below the bulbus and the ventricle was separated from the atrium. The ventricle was cut in pieces, and individual cells were released by gently agitating the muscle pieces. Cell suspension was filtered and kept in isolation solution at 12 ± 1°C in a water bath to prolong cell viability and were used within 8 h. Only elongated cells (spindle shape) with clear cross-striations and without granulation or blebs were used for experiments. All experiments were performed at room temperature (20 ± 1°C).

#### Electrophysiological recordings.

All the experiments described in this article were recorded using the whole cell configuration of the patch-clamp technique settings and properties, as described previously (20). An Axopatch 200B (Axon Instruments, Union City, CA) amplifier was used, controlled by a Pentium PC connected via a Digidata 1322A A/D converter (Axon Instruments), which was also used for data acquisition and analysis using pClamp software (Axon Instruments). Signals were filtered at 2 kHz using an 8-pole Bessel low-pass filter before digitization at 10 kHz and storage. Patch pipettes resistance was typically 1.5–2.5 MΩ when filled with intracellular solution.

#### Recording of I_Ca_.

Experiments were performed using Na^+^- and K^+^-free internal and external solutions to avoid contamination by overlapping ionic currents and to allow the use of a physiological holding potential (16). *I*_Ca_ was recorded during a 250-ms test pulse to 0 mV from a holding potential of −80 mV. Trains of depolarizing pulses were applied at 0.1 Hz, except to investigate CDF (1 Hz).

#### Recording of AP.

APs were evoked by 5-ms subthreshold current steps. Trains of pulses were applied at 0.1 and 1 Hz. AP duration (APD) was measured as the duration from the overshoot to three different percentages of repolarization (25: APD_25_; 50: APD_50_; 90: APD_90_).

#### Whole cell Ca^2+^ transient recordings.

Cardiomyocytes were incubated with the Ca^2+^-sensitive fluorescent indicator Fura-2 AM (5 μM; Molecular Probes, Sunnyvale, CA) for 10 min at room temperature. Fura-2 AM fluorescence was elicited by alternate (every 2 ms) illumination with 340 and 380 nm light obtained using a monochromator (Optoscan Fluorescence System, Cairn Research, Kent, UK) in front of a Xenon excitation lamp. The fluorescence emitted at 510 nm was monitored using a photomultiplier tube (Cairn Research). The ratio (340/380 ratio, ratio unit, RU) was used as an index of [Ca^2+^]_i_. Cells were superfused with control solution (see *Solutions*) and electrically field-stimulated at 0.33 Hz via a pair of platinum electrodes until steady state. Rapid application of caffeine (20 mM) was used to assess SR Ca^2+^ content. pClamp software (Axon Instruments) was used for recordings and analysis.

#### Data analysis.

*I*_Ca_ was measured as the difference between the peak of the inward current and the current at the end of the depolarizing pulse. Because the decay of *I*_Ca_ varied between experimental conditions, the kinetics of *I*_Ca_ inactivation were characterized as the time required for the current to decay to 0.37 of the peak amplitude (T_0.37_), as previously described (20). Therefore, we did not separate monoexponential and biexponential *I*_Ca_. For currents decaying monoexponentially, T_0.37_ is equivalent to the time constant of decay. When the decay was biexponential, T_0.37_ is used as a simple measure to compare the time course of decay in these cells and others. Quantification of inactivation processes (Ca^2+^ and voltage-dependent) was performed as previously described (20) and explained in details in the relevant result section. Frequency-dependent facilitation was analyzed by integrating *I*_Ca_ (pA/ms) during the 250-ms test pulse to obtain total Ca^2+^ influx during the pulse.

#### Solutions.

The isolation solution contained (in mM): 100 NaCl, 10 KCl, 1.2 KH_2_PO_4_, 4 MgSO_4_, 50 taurine, 20 glucose, and 10 HEPES (pH to 6.9 with NaOH). The control bathing solution (Ringer) used for action potential and Ca^2+^ cycling recording contained (in mM): 150 NaCl, 5.4 KCl, 1.5 MgSO_4_, 0.4 NaH_2_PO_4_, 2 CaCl_2_, 10 glucose, and 10 HEPES (pH to 7.7 with NaOH). For *I*_Ca_ recording, cells were locally perfused with Na^+^ and K^+^-free solution, which contained (in mM): 137 TEACl, 6 CsCl, 1 MgCl_2_, 20 HEPES, 10 glucose, 2 CaCl_2_ set to pH 7.6 with TEAOH. In some experiments, CaCl_2_ was replaced with BaCl_2_. The pipette solution for measurement of *I*_Ca_ contained (in mM): 10 TEACl, 125 CsCl, 1 MgCl_2_, 5 Mg-ATP, 2 EGTA, 1 CaCl_2_, 10 HEPES and 0.3 GTPTris (pH to 7.2 with CsOH). In some experiments, EGTA was substituted to 10 mM BAPTA. The free Ca^2+^ concentration in the presence of EGTA is 186 nM and BAPTA is 25 nM [calculated with Maxchelator, Chris Patton, Stanford University, http://maxchelator.stanford.edu, (9)]. We did not adjust for free Ca^2+^ concentration by adding CaCl_2_ since *1*) it will change the osmolarity by more than 6%, which can affect *I*_Ca_ (13, 38); and *2*) this change in basal free Ca^2+^ concentration will not affect SR Ca^2+^ ATPase pump activity (8). Intrapipette solution for AP recording contained (in mM): 139 KCl, 10 NaCl, 0.5 MgCl_2_, 5 Mg-ATP, 0.5 EGTA, 10 HEPES, 0.4 GTP Tris, set to pH 7.2 with KOH. To stimulate β-adrenergic receptors, isoproterenol (Iso, isoproterenol hydrochloride) was applied at 1 μM. All solutions were made using ultrapure water (Millipore, Watford, UK). All solution constituents were reagent grade and purchased from Sigma (Manchester, UK) unless stated otherwise.

#### Statistics.

Data are presented as means ± SE. *P* < 0.05 was taken as significant. Statistical analysis was performed using SigmaStat software. Unpaired *t*-tests or paired *t*-tests were used as appropriate. Student-Newman-Keuls method and Friedman repeated-measures ANOVA on ranks were used to test the effects of stimulation frequency on APD within the same group of cells. Kruskal-Wallis one-way ANOVA on ranks was used to test the effect of isoproterenol on caffeine-induced Ca^2+^ transients.

## RESULTS

### 

#### Characterization of I_Ca_ inactivation in control conditions.

**Fig. 1.**
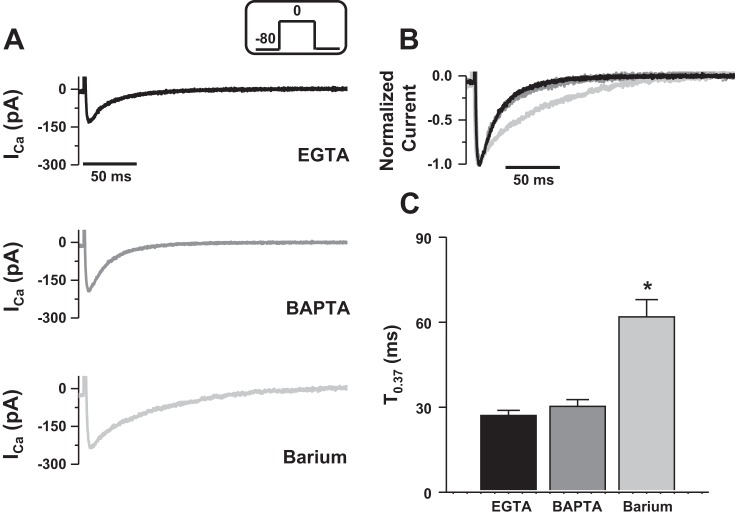
Effect of Ca^2+^ buffers and barium on inactivation of *I*_Ca_. *A*, *top* and *middle*: representative *I*_Ca_ recorded with 2 mM Ca^2+^ as the charge carrier and 2 mM EGTA (black trace) or 10 mM BAPTA (gray trace) in the patch pipette solution. *Bottom*: representative *I*_Ba_ recorded with 2 mM barium as the charge carrier and 10 mM BAPTA in the patch pipette solution (light gray trace). Currents were elicited at 0 mV at a stimulation frequency of 0.1 Hz (voltage step shown in *inset*). *B*: normalized *I*_Ca_ in EGTA (black trace) and BAPTA (gray trace) and normalized *I*_Ba_ (light gray trace). *C*: means ± SE time to decline to 37% of peak *I*_Ca_ (T_0.37_) recorded with EGTA (black bar) or BAPTA (gray bar) and when barium was used as the charge carrier (light gray bar). *Significant difference, *P* < 0.05. Data are from 37 myocytes for EGTA (14 fish), 20 myocytes for BAPTA (8 fish) and 12 myocytes for barium (4 fish).

We first investigated the regulation of LTCCs inactivation by voltage and Ca^2+^ under control conditions to assess whether Ca^2+^ sequestered in the SR participates in global Ca^2+^ transient that activates contraction of rainbow trout ventricular cardiomyocytes. Figure 1*A* shows representative *I*_Ca_ recorded with Ca^2+^ as the charge carrier and either EGTA (black trace, *top*) or BAPTA (gray trace, *middle*) in the patch pipette. To avoid contaminating ionic currents, such as the sodium calcium exchanger current (NCX), cells were locally perfused with a Na^+^ and K^+^-free solution. In presence of EGTA, *I*_Ca_ activated quickly and inactivated with a monoexponential time course, as previously described in fish cardiomyocytes (55). Interestingly, when EGTA was substituted for BAPTA, we observed the same characteristics of *I*_Ca_ activation and inactivation, suggesting that Ca^2+^ is not released by the SR. To quantify the relative contribution of VDI upon CDI, we recorded the current using Ba^2+^ as the charge carrier. In absence of Ca^2+^, CDI no longer takes place and inactivation is exclusively due to VDI. The lower panel of Fig. 1*A* shows representative Ba^2+^ current (*I*_Ba_) recorded with 10 mM BAPTA in the patch pipette solution (light gray trace). Comparison of the time course of the normalized currents (Fig. 1*B*) shows no difference in the decay of the current recorded with EGTA or BAPTA, whereas Ba^2+^ prolonged the time of inactivation, hence, reducing inactivation of the current. To quantify this effect, we calculated the time required for the current to decay to 0.37 of its peak amplitude (T_0.37_, see materials and methods). Mean T_0.37_ ± SE (Fig. 1*C*) show that BAPTA did not increase the decay time constant associated with maximal *I*_Ca_ (T_0.37_: 27.1 ± 1.8 ms with EGTA and 30.3 ± 2.4 ms with BAPTA, NS; *n* = 37 and 20, respectively), while Ba^2+^ slowed it to 61.9 ± 6.1 ms (*P* < 0.05; *n* = 12).

Taken together, these data indicate that under control conditions, *I*_Ca_ does not trigger Ca^2+^ release from the SR of rainbow trout cardiomyocytes and inactivation of Ca^2+^ channels is mainly voltage-dependent, thus contrasting with mammalian cardiomyocytes [e.g., in rat (20) or in rabbit (12)].

#### Rate-dependent changes in I_Ca_ and AP.

**Fig. 2.**
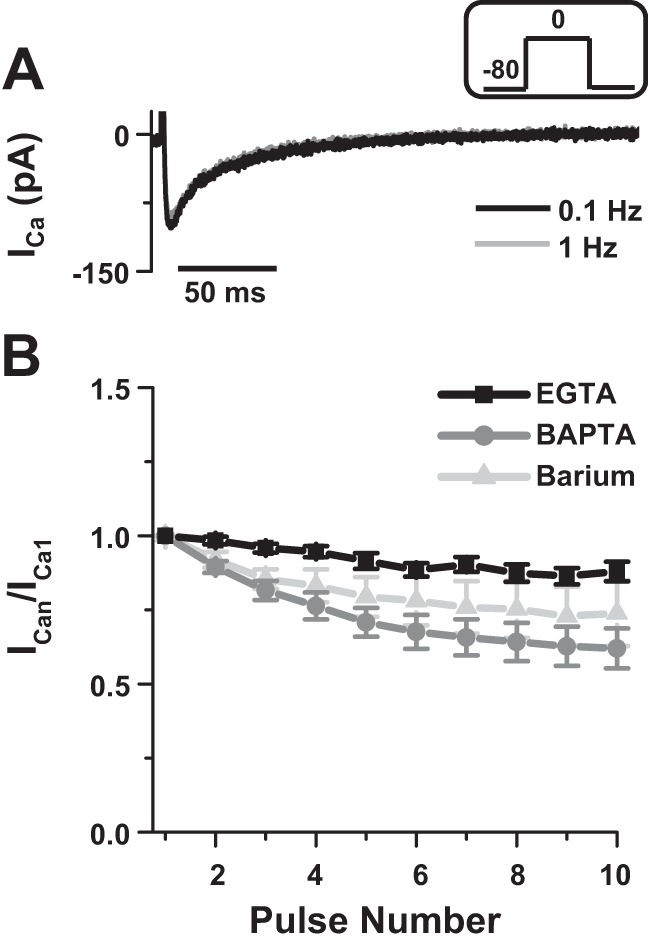
Rate-dependent changes in *I*_Ca_. *A*: representative *I*_Ca_ recorded at 0.1 Hz (black trace, first pulse) and after increasing the stimulation frequency to 1 Hz (gray trace, tenth pulse). Currents were recorded with 2 mM Ca^2+^ as the charge carrier and 2 mM EGTA in the patch pipette solution (voltage step shown in *inset*). Note the absence of a change in *I*_Ca_ shape, indicating no frequency-dependent facilitation. *B*: values are expressed as means ± SE change in *I*_Ca_ area after increasing the stimulation frequency from 0.1 to 1 Hz with 2 mM EGTA (black trace) or 10 mM BAPTA (gray trace) in the patch pipette solution, and when barium (2 mM) was used as the charge carrier (light gray trace). Data are from seven myocytes for EGTA (four fish), nine myocytes for BAPTA (four fish) and 4 myocytes for barium (three fish).

To further comprehend the mechanisms of Ca^2+^ channel regulation, we studied the effect of increasing the pacing rate on the amplitude of *I*_Ca_ to determine whether ventricular cardiomyocytes of rainbow trout displayed CDF. CDF has been described in mammalian cardiomyocytes only and is characterized by an increase in peak *I*_Ca_ amplitude and slowing of inactivation decay during an increase in the pacing rate (see Ref. 16), which was shown to be mainly dependent on SR Ca^2+^ release (21). Figure 2*A* shows representative *I*_Ca_ recorded at 0.1 Hz (black trace) and after increasing the stimulation frequency to 1 Hz (gray trace) with Ca^2+^ as the charge carrier and 2 mM EGTA in the patch pipette solution. *I*_Ca_ amplitude and decay were not significantly changed, indicating an absence of CDF. Figure 2*B* shows average changes in *I*_Ca_ area (pA/ms) after increasing the stimulation frequency from 0.1 to 1 Hz with EGTA (black trace; *n* = 7) or BAPTA (gray trace; *n* = 9) in the patch pipette solution and when Ba^2+^ was used as the charge carrier (light gray trace; *n* = 4). *I*_Ca_ was normalized in response to the first pulse at 1 Hz (after stimulation at 0.1 Hz) and is shown as a function of pulse number. Integrated *I*_Ca_ was not significantly increased with an increase in stimulation frequency, indicating the absence of CDF and supporting the idea that the effect of SR Ca^2+^ release on *I*_Ca_ are limited under basal conditions.

**Fig. 3.**
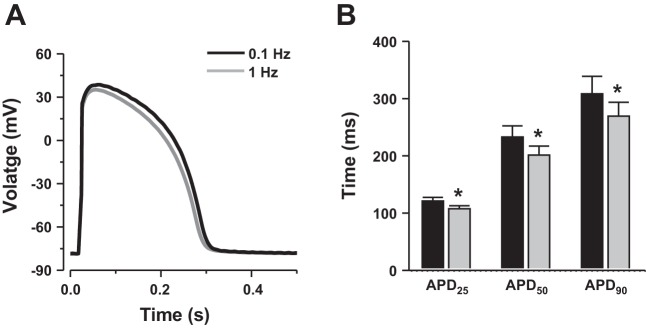
Rate-dependent changes in action potential duration. *A*: representative action potential recorded at 0.1 Hz (black trace) and after increasing the stimulation frequency to 1 Hz (gray trace). Note the reduction in action potential duration. *B*: values are expressed as means ± SE action potential duration at 25, 50, and 90% repolarization (APD_25_, APD_50_, and APD_90_, respectively) recorded at 0.1 Hz (black bars) and 1 Hz (gray bars). *Significant difference, *P* < 0.05. Data are from nine myocytes (six fish).

Because *I*_Ca_ is an important modulator of APD, we investigated the rate-dependent response to an increase in stimulation frequency on the AP. Representative APs recorded at 0.1 Hz (black trace) and 1 Hz (gray trace) are shown in Fig. 3*A*. In agreement with a previous study performed in rainbow trout (27), increasing the pacing rate from 0.1 to 1 Hz induced frequency-dependent changes in APD. At 0.1-Hz stimulation frequency, the APD_25_, APD_50_, and APD_90_ were 116.76 ± 12.07, 247.63 ± 23.70, 332.95 ± 41.41 ms, respectively. At 1 Hz, APD_25_, APD_50_, and APD_90_ significantly decreased to 107.45 ± 8.85, 209.80 ± 22.96, 287.77 ± 38.92 ms, respectively (*n* = 9; *P* < 0.05, Fig. 3*B*). At 0.1 Hz, AP showed a prominent plateau phase that was shorter when the stimulation frequency was increased to 1 Hz. Our results show that the shape of the AP and the negative rate-dependent response to an increase in pacing rate resemble those from other fish species [e.g., from zebrafish; (17)] and large mammalian ventricular cardiomyocytes, including human [e.g., (32)].

Collectively, the absence of CDF and SR CDI in rainbow trout cardiomyocytes indicates that during basal EC coupling, CICR does not occur.

#### Characterization of I_Ca_ inactivation during β-adrenergic stimulation.

**Fig. 4.**
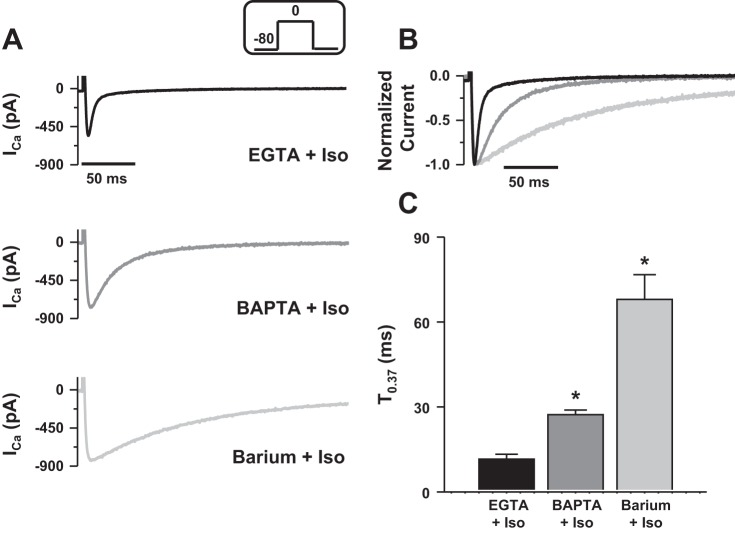
Effect of β-adrenergic stimulation on inactivation of *I*_Ca_. *A, top* and *middle*: representative *I*_Ca_ recorded with 2 mM Ca^2+^ as the charge carrier and 2 mM EGTA (black trace) or 10 mM BAPTA (gray trace) in the patch pipette solution. *Bottom*: representative *I*_Ba_ recorded with 2 mM barium as the charge carrier and 10 mM BAPTA in the patch pipette solution (light gray trace). For each condition, cells were locally perfused with 1 μM isoproterenol (Iso). Currents were elicited at 0 mV at a stimulation frequency of 0.1 Hz (voltage step shown in *inset*). *B*: normalized *I*_Ca_ in EGTA (black trace) and BAPTA (gray trace), and normalized *I*_Ba_ (light gray trace). *C*: values are expressed as means ± SE time to decline to 37% of peak *I*_Ca_ (T_0.37_) recorded with EGTA (black bar) or BAPTA (grey bar) and when barium was used as the charge carrier (light gray bar). *Significant difference, *P* < 0.05. Data are from 18 myocytes for EGTA (4 fish), 12 myocytes for BAPTA (4 fish) and 10 myocytes for barium (4 fish).

In cardiomyocytes, the quantity of Ca^2+^ released by the SR depends on the amplitude of *I*_Ca_, the trigger for release, and the SR Ca^2+^ load (5, 24). Thus, we next tested the possibility that larger *I*_Ca_ was necessary to trigger SR Ca^2+^ release in rainbow trout ventricular cardiomyocytes. We repeated the experiments described above during β-adrenergic stimulation by using 1 μM isoproterenol (Iso), which has previously been shown to cause maximal β-adrenergic receptor stimulation in rainbow trout cardiomyocytes (4). Figure 4*A* shows representative *I*_Ca_ recorded with isoproterenol and EGTA (black trace, *top*) or BAPTA (gray trace, *middle*) in the patch pipette and when Ba^2+^ was used as the charge carrier (light gray trace, *bottom*). In agreement with previous studies (55), application of isoproterenol significantly caused and increase in *I*_Ca_ amplitude that was irrespective of experimental conditions (compare with control, see Fig. 1*A*). However, *I*_Ca_ recorded in EGTA showed rapid and biphasic inactivation, while in BAPTA, inactivation was slowed and remained monophasic (Fig. 4*B*). Accordingly, T_0.37_ was significantly longer when BAPTA was used (T_0.37_: 11.6 ± 1.7 ms in EGTA vs. 27.3 ± 1.6 ms in BAPTA, *P* < 0.05; *n* = 18 and 12, respectively, Fig. 4*C*). When Ba^2+^ was used as the charge carrier, inactivation decay was even slower (T_0.37_: 68.0 ± 8.7 ms; *n* = 12, *P* < 0.05, Fig. 4*C*). These data reveal that during β-adrenergic stimulation, SR CDI participates in *I*_Ca_ inactivation and that Ca^2+^ is released from the SR of ventricular rainbow trout cardiomyocytes (i.e., CICR occurs); hence contrasting with control conditions of this study (see Fig. 1).

#### Assessment of SR Ca^2+^ load.

**Fig. 5.**
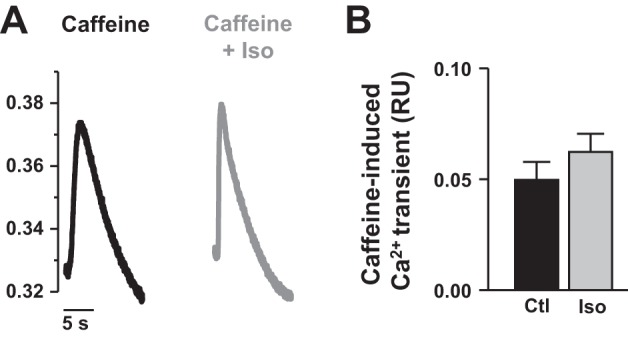
Assessment of SR Ca^2+^ load. *A*: representative caffeine-induced systolic Ca^2+^ transients (20 mM caffeine) recorded in control condition (black trace) and with 1 μM isoproterenol (Iso, gray trace). *B*: means ± SE amplitude of the caffeine-induced Ca^2+^ transient in control (Ctl, black bars) and in the presence of isoproterenol (Iso, gray bars). Data are from 17 myocytes in control (3 fish) and 16 myocytes in isoproterenol (3 fish).

The absence of CICR in control conditions may be due to an absence of Ca^2+^ in the SR. To test this hypothesis, we examined the effect of caffeine pulse (20 mM) on SR Ca^2+^ release under basal conditions and during β-adrenergic stimulation. Figure 5*A* shows representative effect of caffeine application on Fura-2 AM fluorescence in control conditions (black trace) and after superfusion with 1 μM isoproterenol (Iso, gray trace). The amplitude of the caffeine-induced Ca^2+^ transient was used as an index of SR Ca^2+^ content (5). Mean data indicate that isoproterenol did not induce a significant increase in SR Ca^2+^ load (0.050 ± 0.008 RU vs. 0.062 ± 0.008 RU, *P* < 0.05; *n* = 17 and 16, respectively, Fig. 5*B*) but provided evidence that Ca^2+^ is present in the SR in both control conditions and after isoproterenol application. Therefore, the absence of SR Ca^2+^ release during basal EC coupling cannot be attributed to a lack of Ca^2+^ in the SR.

#### SR Ca^2+^ release following sensitization of RyR.

**Fig. 6.**
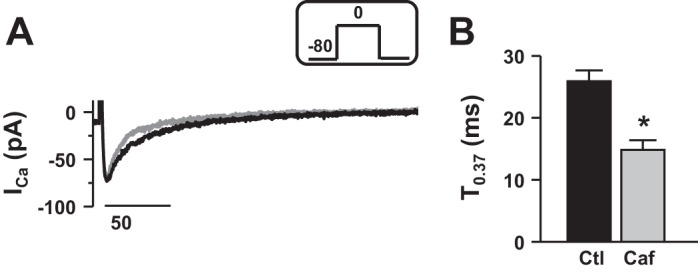
SR Ca^2+^ release following sensitization of ryanodine receptor. *A*: representative *I*_Ca_ recorded in control conditions (black trace) and with 0.5 mM caffeine (gray trace). Currents were elicited at 0 mV at a stimulation frequency of 0.1 Hz (voltage step shown in *inset*). *B*: values are expressed as means ± SE time to decline to 37% of peak *I*_Ca_ (T_0.37_) recorded in control condition with 2 mM EGTA in the patch pipette solution (Ctl, black bar) and with 0.5 mM caffeine (Caf, gray bar). *Significant difference, *P* < 0.05. Data are from 6 myocytes (3 fish).

Given that Ca^2+^ is present in the SR of rainbow trout cardiomyocytes but is not released during basal EC coupling, the next series of experiments were designed to assess whether by increasing the Ca^2+^ sensitivity of the cardiac RyR, CICR can occur. We first checked the effect of 10 μM ryanodine, by incubating the cardiomyocytes for at least 30 min, to further investigate the role of Ca^2+^ from the SR. Ryanodine has no significant effect on T_0.37_ (25.3 ± 1.5 ms in incubated myocytes; *n* = 9 from 4 fish, data not shown, no significance compare to Fig. 1*C*), confirming that no Ca^2+^ release from the SR occurs under control conditions. To increase Ca^2+^ sensitivity of the RyR, we used low concentrations of caffeine [0.5 mM, (37)]. Figure 6*A* shows a representative *I*_Ca_ recorded in control conditions (black trace, 2 mM EGTA) and with 0.5 mM caffeine (gray trace). Compared with the control conditions, application of 0.5 mM caffeine significantly decreased T_0.37_ (from 25.9 ± 1.8 ms in control to 14.9 ± 1.6 ms with caffeine, *n* = 6, *P* < 0.05, Fig. 6*B*) without affecting the amplitude of *I*_Ca_. These data demonstrate that when the Ca^2+^ sensitivity of the RyR is increased, *I*_Ca_ can release Ca^2+^ from the SR, even in basal conditions.

#### Quantification of I_Ca_ inactivation.

To quantify the relative contribution of the components of *I*_Ca_ inactivation in rainbow trout cardiac cardiomyocytes, we measured the fraction of current remaining 20 ms after its peak (*I*_R20_), as in our previous study in rat ventricular cardiomyocytes (20). This time was chosen because the peak of SR Ca^2+^ release occurs at about 5 ms after peak *I*_Ca_, with a time to 90% decay of about 45 ms (50). The fraction of *I*_Ca_ remaining 20 ms after its peak and the proportion of CDI under control conditions and during β-adrenergic stimulation are summarized in Table 1. When *I*_Ca_ is recorded using Ca^2+^ as the charge carrier, *I*_Ca_ inactivation is due to CDI and VDI. When current is recorded with Ba^2+^ as the charge carrier (*I*_Ba_), CDI no longer occurs and inactivation is exclusively due to VDI. Thus, the difference between *I*_Ca_ and *I*_Ba_ represents the fraction of current inactivated by total CDI. By normalizing to *I*_R20Ba_, {[(*I*_R20Ba_ − *I*_Ca R20EGTA_)/I_R20Ba_] × 100}, we estimated that total CDI represents 39% of *I*_Ca_ inactivation in control conditions. To separate SR Ca^2+^ release-induced CDI from total CDI, we further compared *I*_Ca_ recorded in EGTA and BAPTA. Thus, the difference between *I*_R20EGTA_ and *I*_R20BAPTA_ represents the current inactivated by SR-induced CDI. By normalizing to total CDI {[(*I*_R20BAPTA_ − *I*_R20EGTA_)/(*I*_R20Ba_ − *I*_R20EGTA_)] × 100}, we estimated that SR CDI represents 16% of total CDI. Collectively, these data show that under basal conditions, VDI is the major determinant of *I*_Ca_ inactivation in rainbow trout ventricular cardiomyocytes. This differs from mammalian species [e.g., rat (20) and rabbit (12)], where CDI is the prominent inactivation mechanism under basal conditions. In contrast, during β-adrenergic stimulation, inactivation of *I*_Ca_ in the fish cardiomyocyte was switched to a Ca^2+^-dependent mode (65% of total *I*_Ca_ inactivation). SR CDI accounted for nearly half of total CDI (46% *I*_Ca_ inactivation due to SR CDI). These results also demonstrate that in fish cardiomyocytes, CICR is triggered during β-adrenergic stimulation. Interestingly, the proportion of inactivation due to total CDI and SR CDI observed during β-adrenergic stimulation in rainbow trout cardiomyocytes is similar to inactivation of *I*_Ca_ in mammalian species in control conditions.

**Table 1.** Fraction of I_Ca_ remaining 20 ms after its peak and proportion of CDI in control condition and during β-adrenergic stimulation

	Control	Iso
*I*_R20Ba_*	0.72 ± 0.04	0.74 ± 0.04
*I*_R20BAPTA_*	0.49 ± 0.03	0.48 ± 0.02
*I*_R20EGTA_*	0.44 ± 0.02	0.26 ± 0.03
CDI/total inactivation, %†	39	65
SR CDI/Total CDI, %#	16	46

* Values are means ± SE from data in Figs. 1 and 4.

† Calculated as [(*I*_R20Ba_ − *I*_R20EGTA_)/*I*_R20Ba_] × 100.

# Calculated as [(*I*_R20BAPTA_ − *I*_R20EGTA_)/(*I*_R20Ba_ − *I*_R20EGTA_)] × 100. *I*_R20Ba_ indicates Ba^2+^ as charge carrier; *I*_R20BAPTA_, Ca^2+^ as charge carrier and BAPTA in the pipette solution; *I*_R20EGTA_, Ca^2+^ as charge carrier and EGTA in the pipette solution.

CDI, Ca^2+^-dependent inactivation; SR, sarcoplasmic reticulum.

#### I_Ca_ density and SR Ca^2+^ release.

**Fig. 7.**
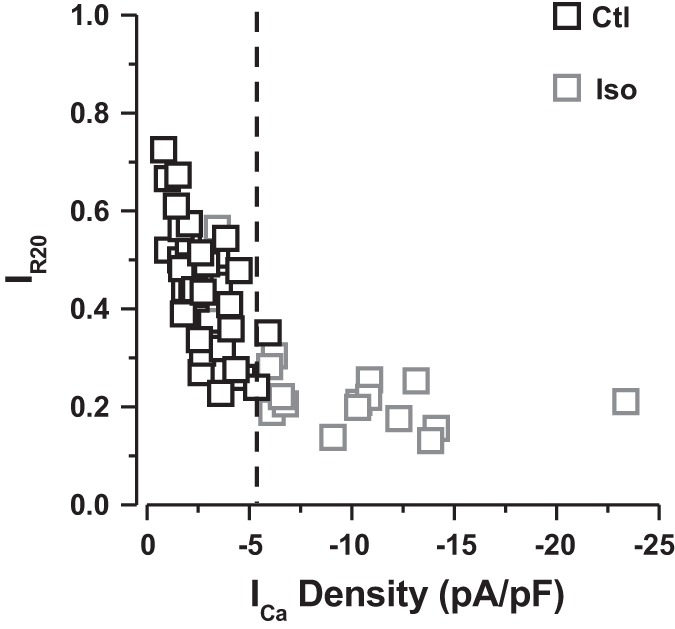
Relationship between *I*_Ca_ density and SR Ca^2+^ release. Values are expressed as means ± SE density of *I*_Ca_ as a function of current remaining 20 ms after its peak (*I*_R20_) recorded in control condition (Ctl, black squares) and during perfusion with 1 μM isoproterenol (Iso, gray squares). Data are from 37 myocytes in control (14 fish) and 18 myocytes in isoproterenol (4 fish).

Finally, we investigated the relation between the density of *I*_Ca_ and the Ca^2+^ release from the SR (measured as *I*_R20_). Figure 7 shows the relation between the density of *I*_Ca_ and *I*_R20_ in control conditions (Ctl, black squares) and during perfusion with 1 μM isoproterenol (Iso, gray squares). Under control conditions (*I*_Ca_ recorded with 2 mM EGTA in pipette solution), *I*_Ca_ density is low and inactivation is slow (*I*_R20_ elevated), indicating an absence of Ca^2+^ release. Indeed, *I*_R20_ decreases linearly as *I*_Ca_ density increases, most probably due to an increase of Ca^2+^ entry via LTCCs, which, in turn, inactivate the channel (see introduction). Perfusion with 1 μM isoproterenol increased *I*_Ca_ density and *I*_Ca_ inactivation (*I*_R20_). *I*_R20_ plateaued at a value (∼0.2), and the relation between *I*_Ca_ density and *I*_Ca_ inactivation is now flat because CICR occurs. A threshold of ∼6 pA/pF is needed for this (dotted line in Fig. 7). Such density of *I*_Ca_ current is obtained mainly under β-adrenergic stimulation.

## DISCUSSION

Our study provides, for the first time, a functional role for the Ca^2+^ stored in the SR of rainbow trout ventricular cardiomyocytes. At rest, SR Ca^2+^ release is not required for normal EC coupling, whereas, during adrenergic stimulation, it plays a significant role in cardiac EC coupling. In addition, our study provides a quantification of inactivation mechanisms of ventricular *I*_Ca_. We show that under control conditions, *I*_Ca_ inactivation is mainly voltage-dependent, while during β-adrenergic stimulation, it shifts to a Ca^2+^-dependent mode with a ratio of SR CDI to total CDI similar to a mammalian system.

### 

#### Experimental approach.

In the present study, we have applied a similar method to that previously described in mammalian cardiomyocytes (20) and used the inactivation of *I*_Ca_ as an index of SR Ca^2+^ release in rainbow trout ventricular myocytes. In cardiomyocytes, there is bidirectional cross-talk between *I*_Ca_ and the SR Ca^2+^ release channel RyR, wherein global Ca^2+^ signaling results from the spatial summation of local Ca^2+^ events occurring in a restricted diffusion space where LTCCs and RyRs colocalized (see Ref. 7). Application of a low concentration of a slow Ca^2+^ buffer (2 mM EGTA) will clamp the bulk of cytosolic Ca^2+^, while allowing the concentration of Ca^2+^ in the restricted diffusion space to change, hence permitting *I*_Ca_ to trigger SR Ca^2+^ release (CICR can occur, and inactivation is rapid).

In contrast, in the presence of a fast Ca^2+^ chelator (10 mM BAPTA), this local Ca^2+^ cycling is prevented (no CICR triggered), such that *I*_Ca_ inactivation is slower (50) and reflects a small rise in local Ca^2+^ concentration close to the mouths of channels due to Ca^2+^ entering via the LTCC only (SR Ca^2+^ release is prevented) (53). Thus, by comparing the inactivation phase of *I*_Ca_ when the current is recorded with either of those two buffers, it is possible to determine whether or not *I*_Ca_ induces SR Ca^2+^ release. In addition, substitution of Ca^2+^ for Ba^2+^ as the charge carrier renders the inactivation solely voltage-dependent, such that it is possible to differentiate VDI from total CDI and calculate the relative contribution of those mechanisms during EC coupling (20).

The presence of exogenous Ca^2+^ buffer and NCX block [important given the relative role in trout myocytes (10)] may interfere with SR Ca^2+^ loading, although it has been shown that 14 mM EGTA in the pipette solution does not significantly alter SR Ca^2+^ load (1), and a recent computer modeling study suggests even an increase (39). Quantification of SR Ca^2+^ load, when exogenous Ca^2+^ buffers are used, are lacking; however, numerous studies in cardiomyocytes from mammals have shown that Ca^2+^ is present in the SR (1, 14, 15, 20, 34, 51, 59) and that there is a functional communication between L-type Ca^2+^ channel and ryanodine receptor.

#### SR Ca^2+^ release is not required for basal EC coupling in fish cardiomyocytes.

In control conditions, inhibition of SR Ca^2+^ release by the fast Ca^2+^ buffer BAPTA did not slow the time of *I*_Ca_ inactivation indicating that *1*) CICR does not occur and *2*) *I*_Ca_ is sufficient to elevate [Ca^2+^]_i_ enough to trigger contraction of rainbow trout cardiomyocytes. These observations validate the current model of most fish cardiac EC coupling suggested in previous studies. They had been mainly attributed to the small dimensions of the fish cardiomyocyte that facilitate the activation of the myofilaments by sarcolemmal Ca^2+^ and also to the limited effect of the SR inhibitor ryanodine on cell contraction (22, 28, 56). Indeed, the long and narrow shape of the fish cardiomyocyte, which, combined with the lack of transverse tubules (invagination of the sarcolemmal membrane in mammalian ventricular myocytes), implies that the SR is exclusively located at the periphery of the fish cell (41). Consequently, the distance of diffusion for SR Ca^2+^ release is similar to that of extracellular Ca^2+^, such that under physiological conditions, SR Ca^2+^ release does not provide any kinetic advantage over sarcolemmal Ca^2+^ (see Ref. 58).

In addition, it was previously demonstrated that both temperature (acute change and acclimation) and heart rate play a key role in the sensitivity to ryanodine in the rainbow trout myocardium (30). In agreement with our findings, the authors observed only minor inhibitory effect of ryanodine on the force of contraction in cardiomyocytes of rainbow trout acclimated at 18°C and paced at 0.6 Hz; hence, suggesting that SR Ca^2+^ is not actively involved in the contraction of cardiomyocytes of those fish under basal conditions (physiological body temperature and hearts rate).

However, the absence of CICR was not due to a lack of Ca^2+^ in the SR, since we observed substantial caffeine-evoked Ca^2+^ transients of magnitude, previously described in rainbow trout cardiomyocytes (27, 47) and mammalian cardiomyocytes (see Ref. 7). Therefore, trout cardiomyocytes store a large amount Ca^2+^ in the SR that is not mobilized during basal EC coupling. The significance of this has been discussed in a recent review (48).

Importantly, we show that when the Ca^2+^ sensitivity of RyR is increased, *I*_Ca_ can release Ca^2+^ from the SR (by using a low dose of caffeine, Fig. 6). In fish cardiomyocytes, few studies have investigated the mechanisms underlying *I*_Ca_ inactivation, and quantitative data are currently lacking. An early study carried out by Vornanen (55) suggested that *I*_Ca_ inactivation in rainbow trout ventricular cardiomyocytes was mainly Ca^2+^-dependent because when Ba^2+^ was used as the charge carrier, the rate of *I*_Ca_ inactivation was significantly reduced. Our quantification refines this observation since in trout cardiomyocytes the decay of *I*_Ca_ was mainly voltage-dependent (61% of total *I*_Ca_ inactivation).

#### SR Ca^2+^ is required for functional EC coupling during β-adrenergic stimulation in fish cardiomyocytes.

In the mammalian heart, the quantity of Ca^2+^ released by the SR depends on the amplitude of *I*_Ca_, the trigger for release, and the SR Ca^2+^ load (5, 24). Thus, we hypothesized that larger *I*_Ca_ may be required to induce SR Ca^2+^ release and tested the effect of β-adrenergic stimulation on inactivation of the Ca^2+^ channel. In fish myocardium, the physiological response to β-adrenergic stimulation is similar to that described for mammals, wherein an increase in cardiac contractility and heart rate occur (46), although the degree of the response is highly species-dependent (55). Interestingly, isoproterenol hastened the time to inactivate *I*_Ca_ when cells were dialyzed with EGTA, but not when SR Ca^2+^ release was inhibited with BAPTA (and Ba^2+^; VDI only). Therefore, our data demonstrate that it is possible to induce CICR in fish cardiomyocytes if *I*_Ca_ is of sufficient amplitude (>6 pA/pF, Fig. 7). Such a situation occurs when the cardiomyocytes are exposed to a stress, and catecholamines are released. Accordingly, more Ca^2+^ would be mobilized to produce a greater contraction, which is achieved by utilization of the Ca^2+^ store in the SR. In such a case, EC coupling in fish myocytes during β-adrenergic stimulation resembles that of adult mammals under control conditions, which rely mainly on SR Ca^2+^. Moreover, the characteristics of *I*_Ca_ inactivation with isoproterenol also resemble those of mammalian cardiomyocytes under control conditions (see Ref. 7). Our data show that *I*_Ca_ inactivation during β-adrenergic stimulation is switched to a prominent Ca^2+^-dependent mode (65% of total *I*_Ca_ inactivation) and, importantly, that SR CDI accounts for nearly half of total CDI (46% due to SR CDI). Interestingly, the proportion of *I*_Ca_ inactivation due to total CDI and SR CDI observed during β-adrenergic stimulation in rainbow trout cardiomyocytes was similar to inactivation of *I*_Ca_ in mammalian species observed under control conditions [e.g., in rat (20)]. Associated with the idea that a larger *I*_Ca_ current was necessary to trigger CICR in fish cardiomyocytes, we further showed that the density of *I*_Ca_ was a critical trigger of SR Ca^2+^ release (Fig. 7). Such high density of *I*_Ca_ is achieved during β-adrenergic stimulation. Below this threshold, the density of the current is not sufficient to induce SR Ca^2+^ release (i.e., CICR). This idea is supported by the observation that a low concentration of caffeine was sufficient to induce CICR, even with low amplitude of the trigger, *I*_Ca_ (above). In mammals, basal *I*_Ca_ density is >6 pA/pF. Therefore, it is tempting to speculate that SR Ca^2+^ release is triggered regardless of the current density (see Ref. 7). The requirement for a larger *I*_Ca_ trigger, or a low caffeine-sensitized ryanodine receptor for CICR in rainbow trout cardiomyocytes may be related to the spatial organization of ryanodine receptors and LTCCs (see Refs. 25 and 48 for recent reviews).

#### Effect of pacing rate on I_Ca_ and APD.

In the current study, we have also investigated the effect of increasing the pacing rate on *I*_Ca_ amplitude and shape and APD. In larger mammals, an increase in cardiac frequency leads to a decrease in APD due to modifications in Ca^2+^ and Na^+^ homeostasis (see Ref. 23) and to a gradual increase in *I*_Ca_ current amplitude and a slowing of inactivation (see Ref. 16). Concomitant with other studies in fish (18, 57), AP of rainbow trout cardiomyocytes displayed a prominent plateau phase with a long duration of about 300 ms. The APD was also decreased when the pacing rate was increased from 0.1 to 1 Hz, as previously described (27). Thus, the AP waveform of rainbow trout ventricular cardiomyocytes and the negative rate-dependent response to an increase in pacing rate closely resemble those from other fish species [e.g., from zebrafish (17)] and from large mammalian species, notably humans (32). However, an increase in the stimulation frequency had no effect on the amplitude and shape of *I*_Ca_, indicating the absence of CDF in rainbow trout ventricular cardiomyocytes, as previously described, but when using a prepulse to −40 mV (27). In mammals, facilitation is reduced by both BAPTA (6, 60) and SR Ca^2+^ release inhibitors (26), reinforcing the idea that SR Ca^2+^ is not released during control conditions in most fish cardiomyocytes.

### Perspectives and Significance

Our study demonstrates that rainbow trout cardiomyocytes utilize the Ca^2+^ stored in the SR upon β-adrenergic stimulation. Thus, our data provide the first evidence to support the hypothesis proposed more than 30 years ago (52) that the Ca^2+^ stored in the SR of trout cardiomyocytes serves as a safety or backup mechanism, released when extra Ca^2+^ is required to increase contractility of the ventricle, such as occurs during stress. An earlier study has shown that cardiac strip in rainbow trout was sensitive to ryanodine and, indeed, adrenaline was used under basal conditions (43). Further studies are required to quantify the respective role of Ca^2+^ from the extracellular fluid and the SR to the Ca^2+^ transient responsible for contraction in trout cardiomyocytes, as it has been recently done in zebrafish (11). It should also be cautioned that these results in rainbow trout may not apply to all fish species, given large differences in behavior and environment. Further studies addressing the role of temperature may provide some insight, even in the same species, since acclimation can change the role of SR (45, 58).

## GRANTS

This study was funded by the Wellcome Trust (UK, Research Career Development Fellowship to F. Brette) and The Biotechnology and Biological Sciences Research Council (UK, Project Grant to H. A. Shiels).

## DISCLOSURES

No conflicts of interest, financial or otherwise, are declared by the authors.

## AUTHOR CONTRIBUTIONS

Author contributions: C.C. and F.B. conception and design of research; C.C., L.S., D.E.W., and F.B. performed experiments; C.C., L.S., and F.B. analyzed data; C.C., L.S., H.A.S., and F.B. interpreted results of experiments; C.C. and F.B. prepared figures; C.C. drafted manuscript; C.C., L.S., D.E.W., H.A.S., and F.B. edited and revised manuscript; C.C., L.S., D.E.W., H.A.S., and F.B. approved final version of manuscript.
